# Recurrent balloon rupture during Transcatheter Aortic Valve Replacement (TAVR) - a challenge to the Hybrid team

**DOI:** 10.1186/1749-8090-10-S1-A316

**Published:** 2015-12-16

**Authors:** Imthiaz Manoly, Andrew Brazier, Ahmed Mustafa, Vasim Farooq, Ragheb IR Hasan, Douglas G Fraser

**Affiliations:** 1 Manchester Heart Centre Central Manchester University Hospitals Manchester, Lancashire, M13 0HX, UK

## Background/Introduction

Transcatheter aortic valve implantation is at present reserved for patients with severe aortic valve stenosis who are high risk for conventional surgery or surgical access is difficult due to heavily calcified aorta (porcelain aorta). However the calcium spikes at the root could pose a problem during balloon valvuloplasty resulting in rupture of the balloon.

## Aims/Objectives

Our objective was to assess patients who have heavy calcification of the aortic valve and aortic root and recommend measures to overcome the difficulty in the deployment of transcatheter valve.

## Method

After careful assessment including computed tomography of the arterial tree, it was decided that the best approach was trans-aortic as the heavy calcification would not allow the trans-femoral or transapical approach feasible. Despite this, there were ruptures of the balloon twice during valvuloplasty which were retrieved at the ascending aorta.

## Results

We were successful in deploying a 26 mm Edwards Sapien XT through trans-aortic approach after two failed attempt due to rupture of the balloon during valvuloplasty. The patient also had an off pump coronary artery bypass grafting to the right coronary artery. The patient was discharged home after a week.

## Discussion/Conclusion

TAVR balloon rupture is an uncommon complication; it could result when the balloon expands against a sharp spike of calcium as it was in this case. The issue of retrieving the balloon could be complicated in such scenarios particularly if the access was through a trans-femoral route. In such a situation the retrieval of the balloon and sheath system would require pulling back the delivery system into the descending aorta and performing a potentially complicated unplanned ilio-femoral vascular exploration. On the other hand, the appearances of sharp spike-like calcifications would favour the trans-aortic approach where retrieval of the system if the balloon ruptures is more straightforward. Pre-operative Computed tomography is very essential in selecting the best access for trans catheter valve.

